# Effects of a virtual reality-based mirror therapy system on upper extremity rehabilitation after stroke: a systematic review and meta-analysis of randomized controlled trials

**DOI:** 10.3389/fneur.2023.1298291

**Published:** 2024-01-08

**Authors:** Ryohei Okamura, Akira Nakashima, Takefumi Moriuchi, Kengo Fujiwara, Kanta Ohno, Toshio Higashi, Kounosuke Tomori

**Affiliations:** ^1^Graduate School of Biomedical Sciences, Nagasaki University, Nagasaki, Japan; ^2^Major of Occupational Therapy, Department of Rehabilitation, School of Health Science, Tokyo University of Technology, Tokyo, Japan

**Keywords:** stroke, mirror therapy, virtual reality, upper extremity, meta-analysis

## Abstract

**Introduction:**

Virtual reality-based mirror therapy (VRMT) has recently attracted attention as a novel and promising approach for treating upper extremity dysfunction in patients with stroke. However, the clinical efficacy of VRMT has not been investigated.

**Methods:**

This study aimed to conduct a meta-analysis to evaluate the effects of VRMT on upper extremity dysfunction in patients with stroke. We screened articles published between January 2010 and July 2022 in PubMed, Scopus, MEDLINE, and Cochrane Central Register of Controlled Trials. Our inclusion criteria focused on randomized controlled trials (RCTs) comparing VRMT groups with control groups (e.g., conventional mirror therapy, occupational therapy, physical therapy, or sham therapy). The outcome measures included the Fugl–Meyer assessment upper extremity test (FMA-UE), the box and block test (BBT), and the manual function test (MFT). Risk of bias was assessed using the Cochrane Collaboration risk-of-bias tool 2.0. We calculated the standardized mean differences (SMD) and 95% confidence intervals (95% CI). The experimental protocol was registered in the PROSPERO database (CRD42022345756).

**Results:**

This study included five RCTs with 148 stroke patients. The meta-analysis showed statistical differences in the results of FMA-UE [SMD = 0.81, 95% CI (0.52, 1.10), *p* < 0.001], BBT [SMD = 0.48, 95% CI (0.16, 0.80), *p* = 0.003], and MFT [SMD = 0.72, 95% CI (0.05, 1.40), *p* = 0.04] between the VRMT and the control groups.

**Discussion:**

VRMT may play a beneficial role in improving upper extremity dysfunction after stroke, especially when combined with conventional rehabilitation. However, there were differences in the type of VRMT, stage of disease, and severity of upper extremity dysfunction. Multiple reports of high-quality RCTs are needed to clarify the effects of VRMT.

**Systematic review registration:**

https://www.crd.york.ac.uk/prospero/, identifier CRD42022345756.

## 1 Introduction

Mirror therapy (MT) is a treatment modality that induces cortical reorganization and promotes plastic changes in the brain without requiring movement of the affected limb (1). MT was initially reported by Ramachandran et al. (2), as a promising intervention for reducing phantom pain in amputees. Since then, it has been used as a therapeutic approach to address upper extremity dysfunction in patients with stroke (3). In a systematic review and meta-analysis conducted by Thieme et al. (4), MT was shown to be effective in improving upper extremity motor function, motor disability, activities of daily living, and pain and is considered to be a complementary treatment to conventional therapy for stroke patients, aiding in their recovery.

The development of innovative technologies has led to a considerable focus on new stroke rehabilitation approaches that utilize virtual reality (VR). VR systems can be categorized into three types: non-immersive, semi-immersive, and immersive (5, 6). Recently, a growing number of intervention studies have used immersive VR with head-mounted displays (HMDs) in patients with stroke (6). These VR systems have been suggested to induce neural plasticity and contribute to functional recovery after stroke (7, 8). Additionally, the VR-based mirror therapy system (VRMT), which applies the concept of MT, is expected to be an effective and innovative treatment method compared with conventional MT (cMT) (9, 10). Several previous studies have reported similarities in brain activity between VRMT and cMT (11–14). These findings indicate that VRMT induces neural plasticity, providing sufficient neurophysiological basis for its clinical application. However, the clinical effects of VRMT have not been investigated.

This review investigated the effects of VRMT on the upper extremities after stroke. We defined VRMT as “synchronized visual feedback of the affected side’s movement with that of the unaffected side.”

## 2 Methods

This review followed the preferred reporting items for systematic reviews and meta-analyses (PRISMA) guidelines (15). The systematic review protocol was registered in PROSPERO (CRD42022345756).

### 2.1 Eligibility criteria

The inclusion criteria were as follows: (1) randomized controlled trials (RCTs) that compared VRMT (based on the definition provided in the previous section) groups with control groups (e.g., cMT, occupational therapy, physical therapy, or sham therapy); (2) primary outcomes of the upper extremities in the adult stroke population; (3) articles published between January 2010 and July 2022; (4) articles published in English. Review articles, case studies, opinion studies, and studies that did not provide detailed descriptions of their procedures were excluded.

### 2.2 Search strategy

We searched the following four scientific databases using online search engines: PubMed, Scopus, MEDLINE, and the Cochrane Central Register of Controlled Trials. The research questions followed the PICOS framework: population (stroke), intervention (VRMT based on the definition provided in the previous section), comparison [control group (e.g., cMT, occupational therapy, physical therapy, sham therapy)], outcomes (upper extremity function), and studies (RCTs) (16). We initially developed search strategies for PubMed, and then adapted them for use in other databases. The following outlines the complete combination of search terms used to search titles and abstracts of potential papers in PubMed, adapted to search other databases:

((stroke[mh])OR(“cerebrovascular disorder”[tw])OR(“brain infarction”[tw])OR(“brain stem infarctions”[tw])OR(lacunar[tw])OR(“brain injury”[tw])OR(“cerebral infarction”[tw])) AND((“mirror therapy”[tw])OR(“mirror visual feedback”[tw])OR(mirror[tw])OR (“mirror training”[tw])OR(“mirror box” [tw]))AND((“virtual rehabilitation”[tw])OR(“virtual reality”[tw])OR(VR[tw])OR(“head mount display”[tw])OR(“head mount”[tw])OR(“head-mounted displays”[tw])OR(“head-mounted”[tw]) OR(HMD[tw])OR(immersive[tw]))

Five authors (RO, AN, TM, KF, and TH) participated in the screening process. Each report was assigned to two authors and screened according to the inclusion and exclusion criteria. First, two authors independently read the title and abstract to exclude irrelevant papers, then they read the full text to determine inclusion. In cases of disagreement, a third author assisted in the judgment.

### 2.3 Methodological quality assessment

Three authors (RO, TM, and KF) participated in the methodological quality assessment process. Each report was assigned to two authors and assessed independently using the Cochrane Collaboration risk-of-bias tool (RoB2.0) (17), which assesses the following sources of bias: (1) randomization process; (2) deviations from intended intervention; (3) missing outcome data; (4) measurement of the outcome; (5) selection of the reported result. Any disagreements were solved by asking the assistance of a third author (TH).

### 2.4 Data extraction

The main outcomes of these studies were the Fugl–Meyer assessment upper extremity test (FMA-UE), the box and block test (BBT), and the motor function test (MFT). The following information was extracted: authors, publication year, study design, country, VR type, VRMT technology, intervention details, sample size, stroke type, age, sex, time since stroke onset, and outcomes. Three authors (RO, TM, and KF) participated in the data extraction process. Each report was assigned to two authors, who extracted the data independently from each other to avoid potential data extraction errors. Any disagreements were solved by a third author (TH).

### 2.5 Statistical analysis

RevMan 5.4 software (RevMan V5.4, Cochrane, London, United Kingdom) was used for the meta-analysis, with the standardized mean difference (SMD) and 95% confidence interval (95% CI) as the statistics of interest. Based on the guidance provided by Cohen, the SMD was interpreted as follows: no effect (SMD = 0), small (SMD = 0.2), medium (SMD = 0.5), and large (SMD ≥0.8) (18). Furthermore, to better understand the effects of each study on the included outcomes, we calculated the mean difference (MD) of the experimental group before and after the intervention and compared it with the minimum clinically important difference (MCID) or minimum detectable change (MDC). We used a random-effects model for the meta-analysis. The post-intervention outcomes were pooled. Statistical heterogeneity was assessed using the *I*^2^ statistic, chi-square test, and *τ*^2^. Funnel plots were used to assess publication bias.

## 3 Results

**Figure 1:**
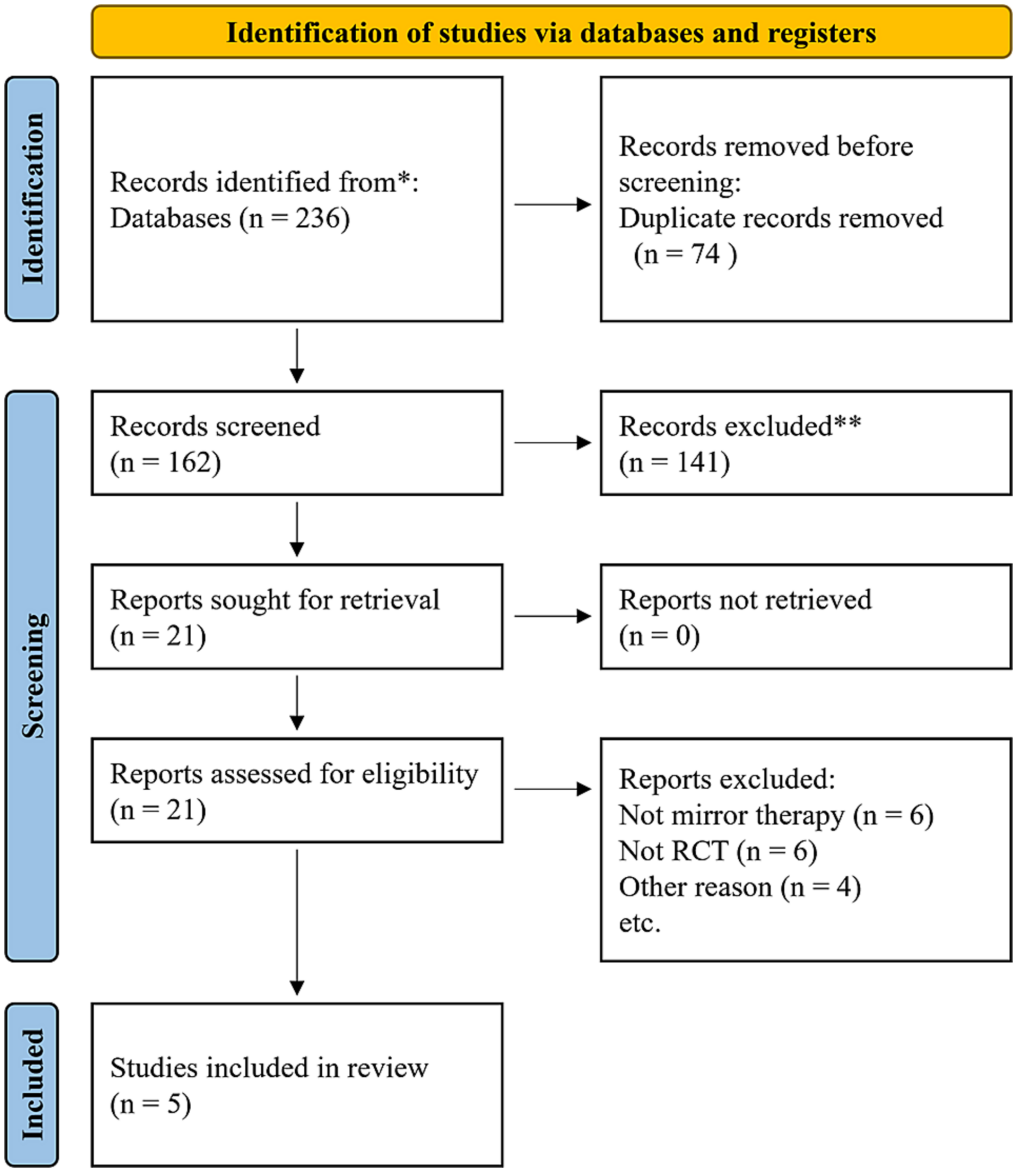
PRISMA flow diagram.

In total, 236 studies were included in the initial search. After removing the duplicates, 162 studies remained. Following screening of titles and abstracts, 21 studies were included for further evaluation. Finally, considering the inclusion and exclusion criteria, the study population consisted of five studies. The detailed search process is illustrated in Figure 1.

### 3.1 Description of the included studies

Table 1 shows the main characteristics of the included five studies. The five studies were conducted in China (19), South Korea (20, 21), and Taiwan (22, 23) over the past 12 years [2012 (21), 2019 (20), 2021 (19, 22), and 2022 (23)]. When combining the data from these studies, a total of 148 stroke patients (mean age of 57.8 ± 4.4 years, 84 males and 64 females) were included. The experimental group consisted of 62 participants (mean age of 57.8 ± 4.4 years), while the control group consisted of 86 participants (mean age of 59.5 ± 2.7 years). Among the included studies, three independently developed and utilized an immersive VRMT system (19, 22, 23). One study employed a VRMT system that combined a wooden box and an LCD monitor (21). One study used a combination of a leap motion controller, monitor, and mirror to construct a VRMT system (20). In all five studies, the experimental group received VRMT combined with conventional rehabilitation approaches such as occupational therapy or physical therapy. The control group was treated with cMT and conventional rehabilitation in three studies (20, 22, 23), a sham therapy and conventional rehabilitation in two studies (20, 21), and only conventional rehabilitation in two studies (19, 23).

**Table 1:** The main characteristics of the included RCTs.

Study	Study design	Country	VR type	VR technology	Intervention and duration of treatment	*N*	Stroke type (CI/ICH)	Age (yrs ± SD)	Gender (M/F)	Diag. time (month or day)	Outcome
Hsu 2022	Single-blind RCT	Taiwan	Immersive	VRMT system (Oculus Rift, Leap Motion Controller, Unity cross-platform game engine)	EG: 30 min VRMT plus 20 min task-specific training, 2/week × 9 weeks CG (cMT): 30 min cMT plus 20 min task-specific training, 2/week × 9 weeks CG (OT): 30 min OT plus 20 min task-specific training, 2/week × 9 weeks	EG: 18 CG (cMT): 17 CG (OT): 17	—	EG: 52.9 ± 11.8 CG (cMT): 56.7 ± 11.5 CG (OT): 56.9 ± 13.0	EG: 8/10 CG (cMT): 7/10 CG (OT): 5/12	EG: 30.7 ± 21.1 CG (cMT): 39.8 ± 28.8 CG (OT): 38.1 ± 26.6	FMA-UE SWM MAL MAS BBT
Mekbib 2021	Single-blind RCT	China	Immersive	MNVR-Rehab (HTC Vive HMD, Leap Motion, HTC Vive tracking stations, ALIENWARE high graphics laptop, Unity 3D game engine)	EG: 60 min VRMT plus 60 min OT, 4/week × 2 weeks CG: 120 min OT, 4/week × 2 weeks	EG: 12 CG: 11	EG: 9/3 CG: 8/3	EG: 52.17 ± 13.26 CG: 61.00 ± 7.69	EG: 9/3 CG: 8/3	EG: 36.92 ± 22.04(days) CG: 39.36 ± 18.08(days)	FMA-UE BI rs-fMRI
Lin 2021	Single-blind RCT	Taiwan	Immersive	VRMT system (Oculus Rift, Leap Motion Controller, Unity cross-platform game engine)	EG: 30 min VRMT plus 20 min motor task-specific training, 2/week × 9 weeks CG: 30 min cMT plus 20 min motor task-specific training, 2/week × 9 weeks	EG: 9 CG: 9	—	EG: 49.7 ± 13.4 CG: 58.8 ± 9.6	EG: 7/2 CG: 6/3	EG: 42.2 ± 21.3 CG: 48.2 ± 32.4	FMA-UE
Choi 2019	Prospective RCT	Korea	Non-immersive	GR mirror therapy (Leap motion controller, a monitor, a mirror, and a Leap Motion App Home)	EG/CG (cMT)/CG (Sham): All 3 groups of therapy: 30 min, 3/week × 5 weeks, plus PT	EG: 12 CG (cMT): 12 CG (Sham): 12	—	EG: 58.00 ± 15.15 CG (cMT): 59.58 ± 11.87 CG (Sham): 59.33 ± 13.63	EG: 7/5 CG (cMT): 7/5 CG (Sham): 9/3	EG: 28.91 ± 15.80 CG (cMT): 26.33 ± 15.51 CG (Sham): 29.00 ± 19.21	MFT NDS SF-8
In 2012	Prospective RCT	Korea	Non-immersive	Virtual reality reflection equipment (Wooden box and an LCD monitor)	EG: 30 min VRMT plus Conventional therapy, 5/week × 4 weeks CG (Sham): 30 min Sham training plus Conventional therapy, 5/week × 4 weeks	EG: 11 CG: 8	EG: 7/4 CG: 3/5	EG: 63.45 ± 11.78 CG: 64.50 ± 12.69	EG: 7/4 CG: 4/4	EG: 14.00 ± 4.88 CG: 12.75 ± 6.78	FMA-UE MAS MFT BBT JTHFT

RCT, randomized controlled trial; VR, virtual reality; VRMT, virtual reality-based mirror therapy; HMD, head-mounted display; GR, gesture recognition; EG, experimental group; CG, control group; cMT, conventional mirror therapy; OT, occupational therapy; *N*, sample size, CI, cerebral infarction; ICH, intracranial hemorrhage; SD, standard deviation; M, male; F, female; Diag. time, time from diagnosis; FMA-UE, Fugl–Meyer assessment upper extremity; SWM, Semmes–Weinstein monofilament; MAL, motor activity log; MAS, modified Ashworth scale; BBT, box and block test; BI, Barthel index; rs-fMRI, resting-state functional magnetic resonance; MFT, manual function test; NDS, Neck discomfort score; SF-8, short-form 8; JTHFT, Jebsen–Taylor hand function test.

### 3.2 Methodological quality assessment

**Figure 2:**
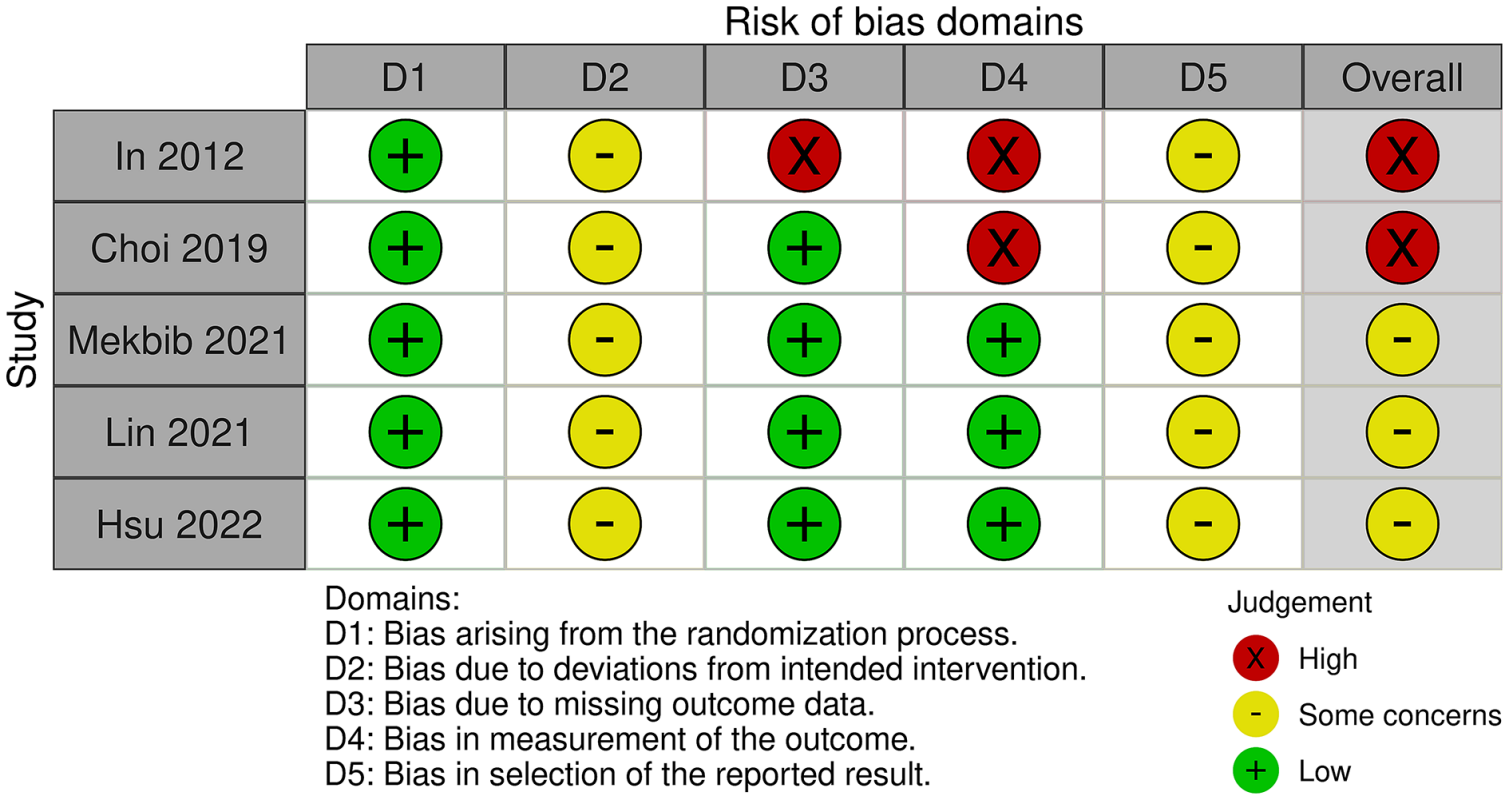
Summary of methodological quality.

**Figure 3:**
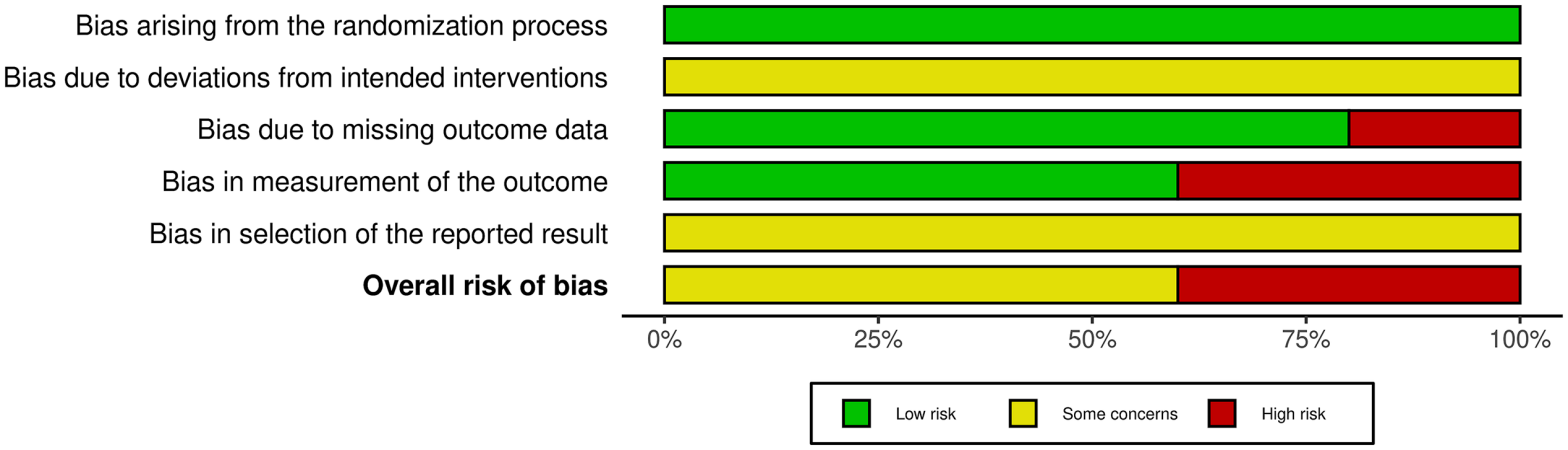
Graph indicating methodological quality.

Five studies were evaluated based on the randomization process, deviations from the intended intervention, missing outcome data, measurement of the outcome, and selection of reported results. In conclusion, two studies had a high risk of bias and three studies had a some concerns (Figures 2, 3). All five studies employed randomization and allocation concealment methods, with one describing a specific randomization method and four describing both a specific randomization method and allocation concealment. There is a possibility that ITT analysis was not conducted in all five studies, raising concerns about its potential impact on the study results. Additionally, in three out of the five studies, there were no descriptions regarding the presence or absence of deviations and their reasons, making it impossible to obtain relevant information. As a result, there were concerns about deviations from the intended intervention in all five studies. While three studies explicitly mentioned the application of blinded assessment, the remaining two studies had a diagnostic detection bias. We were unable to obtain research protocols or plans for any of the five studies. Therefore, we were concerned about the risk of bias in the selection of reported outcomes.

### 3.3 Outcomes measures

Four studies that utilized the FMA-UE to evaluate upper extremity function (19, 21–23), two studies that used the BBT (21, 23), and two studies that employed the MFT (20, 21). Other outcomes measured included the modified Ashworth scale (MAS) (21, 23), Jebsen–Taylor hand function test (JTHFT) (21), Semmes–Weinstein monofilament (SWM) (23), motor activity log (MAL) (23), Neck discomfort score (NDS) (20), short-form 8 (SF-8) (20), and Barthel index (BI) (19).

### 3.4 Fugl–Meyer assessment upper extremities

**Figure 4:**
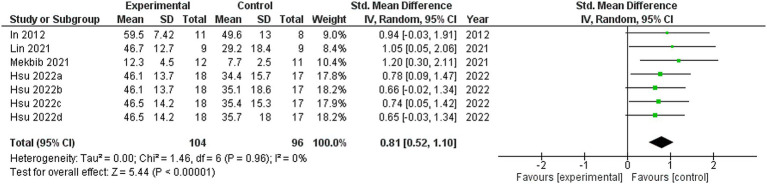
Standardized mean difference (SMD) and overall effect size (95% CI) in meta-analysis of Fugl–Meyer assessment upper extremity tests (FMA-UE): (a) virtual reality-based mirror therapy (VRMT) group versus conventional mirror therapy (cMT) group (9 weeks), (b) VRMT group versus occupational therapy (OT) group (9 weeks), (c) VRMT group versus cMT group (12 weeks), (d) VRMT group versus OT group (12 weeks).

**Figure 5:**
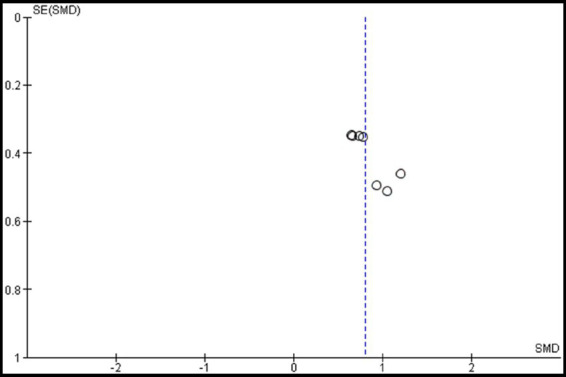
Funnel plot of Fugl–Meyer assessment upper extremity tests.

Four studies were included in this meta-analysis on the intervention effects of VRMT on FMA-UE (19, 21–23). Figure 4 presents the pooled results and forest plots. A study by Lin et al. (22) had reported significant improvements in a group receiving a combination of VRMT and regular motor task-specific training compared to a group receiving a combination of cMT and regular motor task-specific training (21). Hsu et al. (23) included two control groups (cMT and occupational therapy) and conducted a follow-up assessment after 12 weeks. In a study by Hsu et al. (23), significant improvements were reported in the group receiving a combination of VRMT and task-specific training compared with the group receiving cMT and task-specific training in both the post-intervention assessment (9 weeks) and follow-up assessment (12 weeks). In a study by Mekbib et al. (19), the group receiving combined VRMT and occupational therapy reported significant improvements compared to the control group receiving occupational therapy alone. The mean difference in FMA-UE pre- and post-intervention in the experimental group was 3.8 points (9 weeks), 4.2 points (12 weeks) (23), 3.3 points (22), 10.36 points (21) and 3.0 points (19). The integrated results for FMA-UE showed a large overall effect size [SMD = 0.81; 95% CI (0.52, 1.10); *p* < 0.001] favoring the VRMT group compared to the control groups. No significant heterogeneity was observed (*I*^2^ = 0%). The evaluation of publication bias using a funnel plot did not show asymmetry; however, the limited number of included studies was a concern (Figure 5).

### 3.5 Box and block test

**Figure 6:**
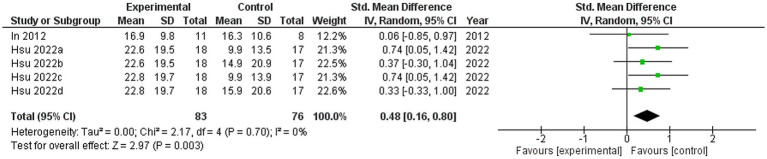
Standardized mean difference (SMD) and overall effect size (95% CI) in meta-analysis of box and block tests (BBT): (a) virtual reality-based mirror therapy (VRMT) group versus conventional mirror therapy (cMT) group (9 weeks), (b) VRMT group versus occupational therapy (OT) group (9 weeks), (c) VRMT group versus cMT group (12 weeks), (d) VRMT group versus OT group (12 weeks).

**Figure 7:**
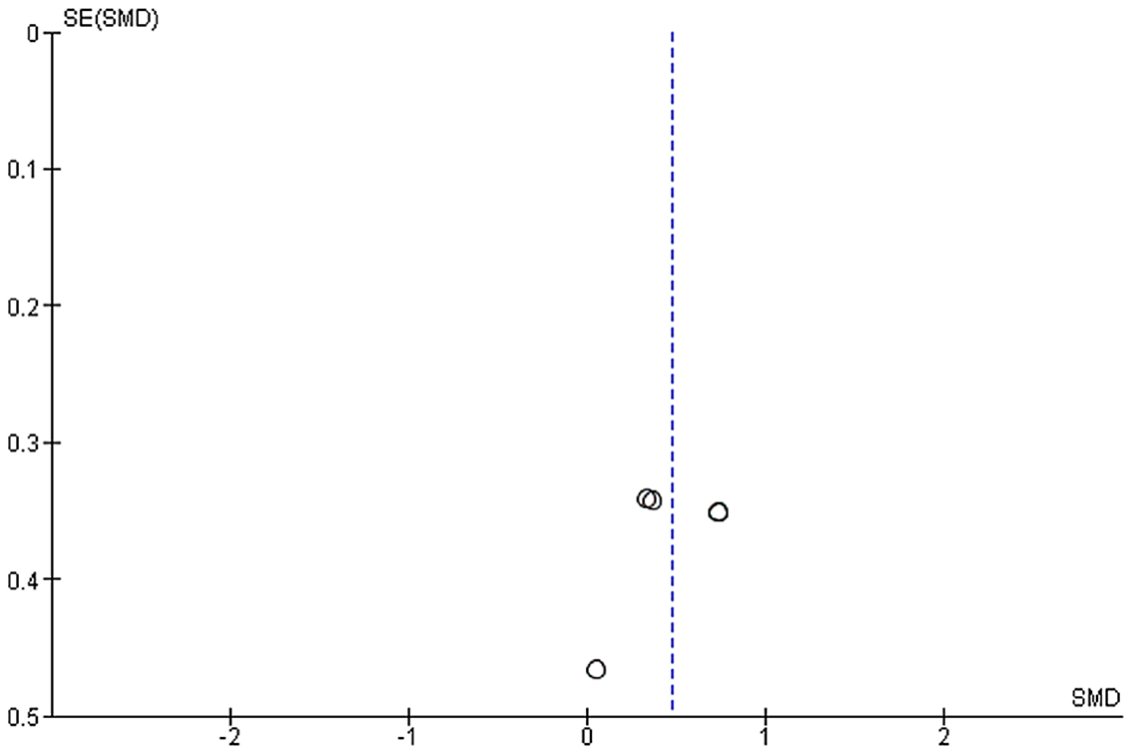
Funnel plot of box and block tests (BBT).

Two studies were included in this meta-analysis to examine the effects of VRMT on BBT (21, 23). The pooled results and forest plots are shown in Figure 6. Hsu et al. (23) reported significant improvements in the group receiving VRMT combined with task-specific training compared to the group receiving cMT combined with task-specific training at both post-intervention (9 weeks) and follow-up assessments (12 weeks) (23). The MD on the BBT pre- and post-intervention in the experimental group were 2.9 points (9 weeks), 3.1 points (12 weeks), respectively (23). and 2.0 points (21). The integrated results for BBT showed a medium overall effect size in favor of the VRMT group compared to the control group [SMD = 0.48; 95% CI (0.16, 0.80); *p* = 0.003]. No significant heterogeneity was observed (*I*^2^ = 0%). Evaluation of publication bias using a funnel plot did not indicate any asymmetry; however, concerns remain regarding the limited number of included studies (Figure 7).

### 3.6 Manual function test

**Figure 8:**
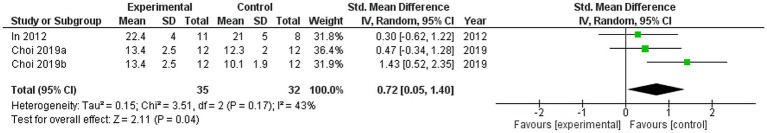
Standardized mean difference and overall effect size (95% CI) in meta-analysis of manual function tests (MFT): (a) virtual reality-based mirror therapy (VRMT) group versus mirror therapy (MT) group, (b) VRMT group versus control group.

**Figure 9:**
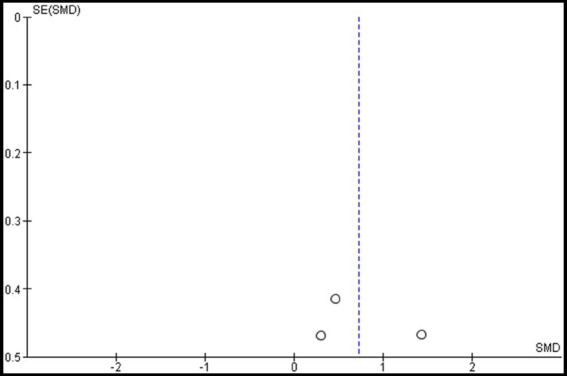
Funnel plot of manual function tests (MFT).

Two studies were included in the meta-analysis to examine the effects of VRMT on MFT (20, 21). The pooled results and forest plots are shown in Figure 8. Choi et al. (20) included two control groups (cMT and control). The study conducted by Choi et al. (20) demonstrated significant improvement when comparing a group receiving a combination of VRMT and PT to a control group receiving a combination of sham therapy and PT. The pre- and post-intervention MD values in the experimental group were 4.5 points (20) and 3.6 points (21). The integrated results for MFT showed a significant overall effect size in favor of the VRMT group compared to the control group [SMD = 0.72; 95% CI (0.05, 1.40); *p* = 0.04]. Moderate statistical heterogeneity was observed based on the *I*^2^ value (*I*^2^ = 43%). However, considering the results of *τ*^2^ and *χ*^2^, it is likely that this heterogeneity was due to chance [*τ*^2^ = 0.15, *χ*^2^ = 3.51 (df = 2, *p* = 0.17)]. Evaluation of publication bias using a funnel plot did not indicate any asymmetry; however, there was concern regarding the limited number of included studies (Figure 9).

## 4 Discussion

This study aimed to investigate the effects of VRMT on upper-extremity dysfunction in patients with stroke based on a meta-analysis of five RCTs. Our findings indicate that VRMT is effective in improving upper extremity dysfunction in these patients. Specifically, large effect sizes were observed for FMA-UE (19, (21–23) and moderate effect sizes were observed for BBT (21, 23) and MFT (20, 21) in the group combined with VRMT. To conduct a comprehensive analysis of our results, we will address them, including the limitations, to ensure a thorough examination.

First, it is important to acknowledge the variation in the VRMT systems used in the five studies included in this meta-analysis. Therefore, comparing and establishing unified criteria for these systems may be challenging. However, despite these variations, the overall effectiveness of systems that apply cMT to VR was demonstrated, suggesting that, regardless of the type of VRMT, systems that apply cMT to VR have the potential to supplement conventional rehabilitation, such as occupational therapy and physical therapy. This finding is consistent with those of previous studies reporting that VR may be beneficial as a supplementary therapy to improve upper extremity function (6). Reviewing the individual studies, three studies used VRMT systems with immersive VR, and most reported large effect sizes (19, 22, 23). Previous studies have suggested that immersive virtual reality produces greater beneficial effects on upper extremity recovery in adult patients with stroke (24). In fact, advantages of VRMT using immersive VR include the following: (1) the possibility of creating more convincing “brain illusions” in a mixture of real and virtual objects because of full immersion, (2) the possibility of observing mirror images in the most natural position for stroke patients, and (3) the possibility of removing noise from the clinical environment, allowing for greater concentration (25). A prerequisite for this advantage is the embodiment of the hand in immersive VR, for which senses of self-location, agency, and ownership are important (25). In addition, recent research has developed a novel and affordable method for texturing virtual hands from individually captured photographs and has reported systems that can create more realistic and personalized virtual hands (26). The use of state-of-the-art technology may increase the effects of VRMT on upper extremity dysfunction after stroke.

Next, the results of this study demonstrated that combining VRMT with conventional rehabilitation facilitates improvement in upper extremity function, as assessed by FMA-UE, BBT, and MFT. However, we must mention that in the included studies, the patients had different periods since diagnosis. Four studies included patients with chronic stroke (20–23), and one study included patients with subacute stroke (19). The variability in the time since diagnosis may impact the study outcomes.

To better understand the results of individual studies, the MD for each outcome before and after the intervention in the experimental group was calculated. Hsu et al. (23) reported an improvement of 3.8 points (9 weeks), 4.2 points (12 weeks) (23), Mekbib et al. (19) 3.0 points, and Lin et al. (22) 3.3 points. These results are below the improvements of 4.25–7.25 points proposed as MCID for FMA-UE in a previous study investigating chronic stroke patients (27). In contrast, In et al. (21) reported an improvement of 10.36 points in MD between pre- and post-intervention FMA-UE in the experimental group. This result exceeds the MCID for FMA-UE, indicating a large effect. However, our methodological quality assessment showed that the study design used by In et al. (21) had the highest risk of bias. Specifically, we detected a high risk of bias in missing outcome data, and outcome measurement. Thus, our results suggest that the effects of VRMT on improving upper extremity function in patients with chronic stroke, as assessed by FMA-UE, may show clinically limited improvements.

In a study of patients with subacute stroke, Mekbib et al. (19) reported a 3.0-point improvement in MD between pre- and post-intervention FMA-UE in the experimental group. This result is below the improvement of 12.4 points proposed as the MCID for FMA-UE in a previous study of patients with subacute stroke (28). This may be because the study by Mekbib et al. (19) had an FMA-UE of 9.25 ± 3.84 points for patients in the experimental group at the beginning of the intervention, and the severity of upper extremity function was more severe than in previous studies reporting MCID (28). In other words, in stroke patients with severe upper extremity dysfunction in the subacute phase, the VRMT group showed significant improvement in FMA-UE compared with the control group, suggesting that this may be a clinically meaningful intervention. Surprisingly, they also reported that the VRMT group showed significant improvement in the Barthel Index, which assesses the activities of daily living, compared to the control group. This result may be explained by the fact that VRMT is based on the concept of cMT, which can increase cortical excitability without moving the affected upper extremity (12, 14, 29, 30). VR systems specifically designed for rehabilitation are more effective than those designed for recreational gaming in improving upper-extremity dysfunction after stroke (31). In short, the study by Mekbib et al. (19) is a clinically interesting and important finding that strongly underscores the need for further investigation into the effects of VRMT in patients with stroke having severe upper extremity dysfunction in the subacute phase.

Thirdly, it is essential to comprehend the characteristics of stroke patients targeted in the five studies and recognize that the results of the meta-analysis may not be applicable to all stroke patients. The inclusion criteria in these studies encompassed a wide range of symptoms in upper extremity function, from mild to severe, based on the FMA-UE (32). Additionally, specific requirements for Mini-Mental State Examination scores (MMSE) included values of 16 or above, 21 or above, or 24 or above, indicating the absence of significant cognitive impairment. Other criteria involved the absence of visual or auditory issues, hemispatial neglect, apraxia, aphasia, and orthopedic surgical conditions. Therefore, there is a potential limitation in the applicability of VRMT to stroke patients who do not meet these specified criteria. To analyze what kind of stroke patients VRMT can be adapted, it would be necessary to conduct subgroup analyses based on factors such as the time since onset, severity, stroke types. However, due to the limited number of included studies in this investigation, such subgroup analyses could not be performed. Therefore, in this study, we cannot specifically mention which stroke patients may potentially benefit more from VRMT, given the limited number of included studies. Very few studies have utilized the BBT and MFT as outcome measures. Furthermore, it was not possible to determine the MCID or MCD for these outcomes, preventing a comprehensive analysis of individual studies. In recent years, many studies on upper extremity function after stroke have used FMA-UE as a primary outcome measure (33). Therefore, future research on the effects of VRMT should consider using FMA-UE as the primary outcome measure. Overall, our results indicate a scarcity of evidence regarding the effects of VRMT on post-stroke upper extremity impairments.

## 5 Conclusion

Based on our investigation, the use of VRMT, when combined with conventional rehabilitation, may be effective in improving upper extremity function after stroke. However, there are a limited number of eligible studies, variability in the stage and severity of stroke progression, and different types of VRMT. As a result, clinical efficacy may be constrained. In future studies aimed at exploring the effects of VRMT, the use of a standardized VRMT system, control of the time since stroke onset and severity of upper extremity, and the incorporation of high-quality RCTs should be considered.

## Data availability statement

The original contributions presented in the study are included in the article/supplementary material, further inquiries can be directed to the corresponding author.

## Author contributions

RO: Writing – original draft. AN: Investigation, Writing – review & editing. TM: Investigation, Writing – review & editing. KF: Investigation, Writing – review & editing. KO: Methodology, Supervision, Writing – review & editing. TH: Project administration, Writing – review & editing. KT: Methodology, Supervision, Writing – review & editing.
